# A 19-Nucleotide Insertion in the Leader Sequence of Avian Leukosis Virus Subgroup J Contributes to Its Replication *in Vitro* but Is Not Related to Its Pathogenicity *in Vivo*

**DOI:** 10.1371/journal.pone.0084797

**Published:** 2014-01-20

**Authors:** Xiaolin Ji, Qi Wang, Xiaofei Li, Xiaole Qi, Yongqiang Wang, Honglei Gao, Yulong Gao, Xiaomei Wang

**Affiliations:** Division of Avian Infectious Diseases, State Key Laboratory of Veterinary Biotechnology, Harbin Veterinary Research Institute, Chinese Academy of Agricultural Sciences, Harbin, China; University of Pittsburgh, United States of America

## Abstract

Subgroup J avian leukosis virus (ALV-J) was first isolated from meat-type chickens that had developed myeloid leukosis and since 2008, ALV-J infections in chickens have become widespread in China. A comparison of the sequence of ALV-J epidemic isolates with HPRS-103, the ALV-J prototype virus, revealed several distinct features, one of which is a 19-nucleotide (nt) insertion in the leader sequence. To determine the role of the 19-nt insertion in ALV-J pathogenicity, a pair of viruses were constructed and rescued. The first virus was an ALV-J Chinese isolate (designated rSD1009) containing the 19-nt insertion in its leader sequence. The second virus was a clone, in which the leader sequence had a deleted 19-nt sequence (designated rSD1009△19). Compared with rSD1009△19, rSD1009 displayed a moderate growth advantage *in vitro*. However, no differences were demonstrated in either viral replication or oncogenicity between the two rescued viruses in chickens. These results indicated that the 19-nt insertion contributed to ALV-J replication *in vitro* but was not related to its pathogenicity *in vivo*.

## Introduction

Avian leukosis viruses (ALVs) constitute a species belonging to the family *Retroviridae*, subfamily *Orthoretrovirinae*, and genus *Alpharetrovirus.* ALVs that infect chickens can be classified into six subgroups (A, B, C, D, E, and J) based on their host ranges, viral cross-neutralization patterns, and viral envelope interference [1], [2]. They can also be divided into exogenous (subgroups A, B, C, D, and J) and endogenous (subgroup E) groups according to the mode of transmission [1]. In China, ALV-J-associated myeloid leukosis in chickens was first reported in 1999 [3] and has since become widespread and responsible for the spread of disease among flocks in many parts of the country and subsequently has caused enormous economic losses to the poultry industry. The morbidity rates for some chicken flocks due to ALV-J infections have reportedly reached 60% and mortality rates for some flocks may be more than 20%. ALV-J infections can induce formation of various tumors and have caused production problems in commercial laying hens and local chicken breeds throughout China [4], [5], [6].

ALV-J has an overall structure of a typical slowly transforming replication-competent ALV: a 5′ long terminal repeat (LTR) leader *gag-pol*-*env* sequence followed by a 3′ LTR [7]. The leader sequence has two unique ATG codons upstream of the conserved ATG codon that introduces the large open reading frame encoding the Gag protein, which is composed of several subunits that form the internal structural components of the viral core [8]. Further along, the leader sequence contains cis-acting sequences and essential transcriptional control elements that are required for viral gene expression [9]. The *gag*, *pol*, and *env* genes encode Gag (a group-specific antigen), a reverse transcriptase integrase, and the envelope glycoproteins, respectively [10], [11].

In recent years, comparisons of the sequences of ALV-J epidemic isolates with HPRS-103, the ALV-J prototype virus, have revealed several distinct features related to its pathogenicity. For example, a 205-nucleotide (nt) deletion and E element in the ALV-J genome occurs naturally and contributes to its pathogenicity [9], [12]. Recently, we analyzed the ALV-J sequence and determined that another distinct feature is a 19-nt insertion in the leader sequence. The question of whether this 19-nt insertion is related to pathogenicity has become an interesting possibility. Reverse genetics was used to produce a wild-type virus with the 19-nt insertion and a variant virus in which the 19-nt sequence was deleted. We rescued the viruses and simultaneously investigated their replication and pathogenicity abilities both *in vitro* and *in vivo*.

## Materials and Methods

### Ethics Statement

All animal experiments were approved by the ethical review board of Harbin Veterinary Research Institute (HVRI) of the Chinese Academy of Agricultural Sciences (CAAS) and performed in accordance with approved animal care guidelines and protocols. The animal Ethics Committee approval number is Heilongjiang-SYXK-2006–032.

### Cell culture and viral isolation

DF-1 cells (ATCC accession number: CRL-12203) were grown in Dulbecco's modified Eagle's medium (DMEM; Invitrogen, Shanghai, China) supplemented with 10% fetal bovine serum and 100 µg/ml of penicillin and streptomycin. Tumors from chickens raised in Shandong province, China [13], were collected aseptically from clinical samples with spontaneous infections and identified as erythroblastosis. Tumor homogenate prepared in serum-free DMEM was lysed by freezing and thawing three times and then the supernatant was purified by low-speed centrifugation and 0.22-mm-pore-size cellulose–acetate filtration. The supernatant was inoculated into a continuous line of chicken fibroblasts (DF-1 cells) and the viruses were then harvested from log-phase culture supernatant and stored at −70°C for further analysis.

### Construction of the rSD1009 and rSD1009△19 infectious clones

Proviral DNA was extracted from SD1009-infected DF-1 cells for use as a template for polymerase chain reaction (PCR) amplification. The SD1009 genome was cleaved into three fragments using the *Xba*I and *Cla*I restriction enzymes, which were amplified separately. The primer sets used to amplify fragment I were 5′-CCCGGCCGTGTAGTGTTATGCAATACTCTTATGTAACGATGAAAC-3′ (upstream, position 1–37)

and 5′-GGCCATTTTCATGTCTAGATT-3′ (downstream, position 3600–3620). The primer sets used to amplify fragment II were 5′-GGCGAGGGAATGGAATCT-3′ (upstream, position 3587–3608) and 5′-GCGCCAGGAGTAAGAAATCGATG-3′ (downstream, position 6633–6655). The primer sets used to amplify fragment III were 5′-CGAGCAGCCATCGATTTCTTACTC-3′ (upstream, position 6625–6648) and

**Figure 1:**
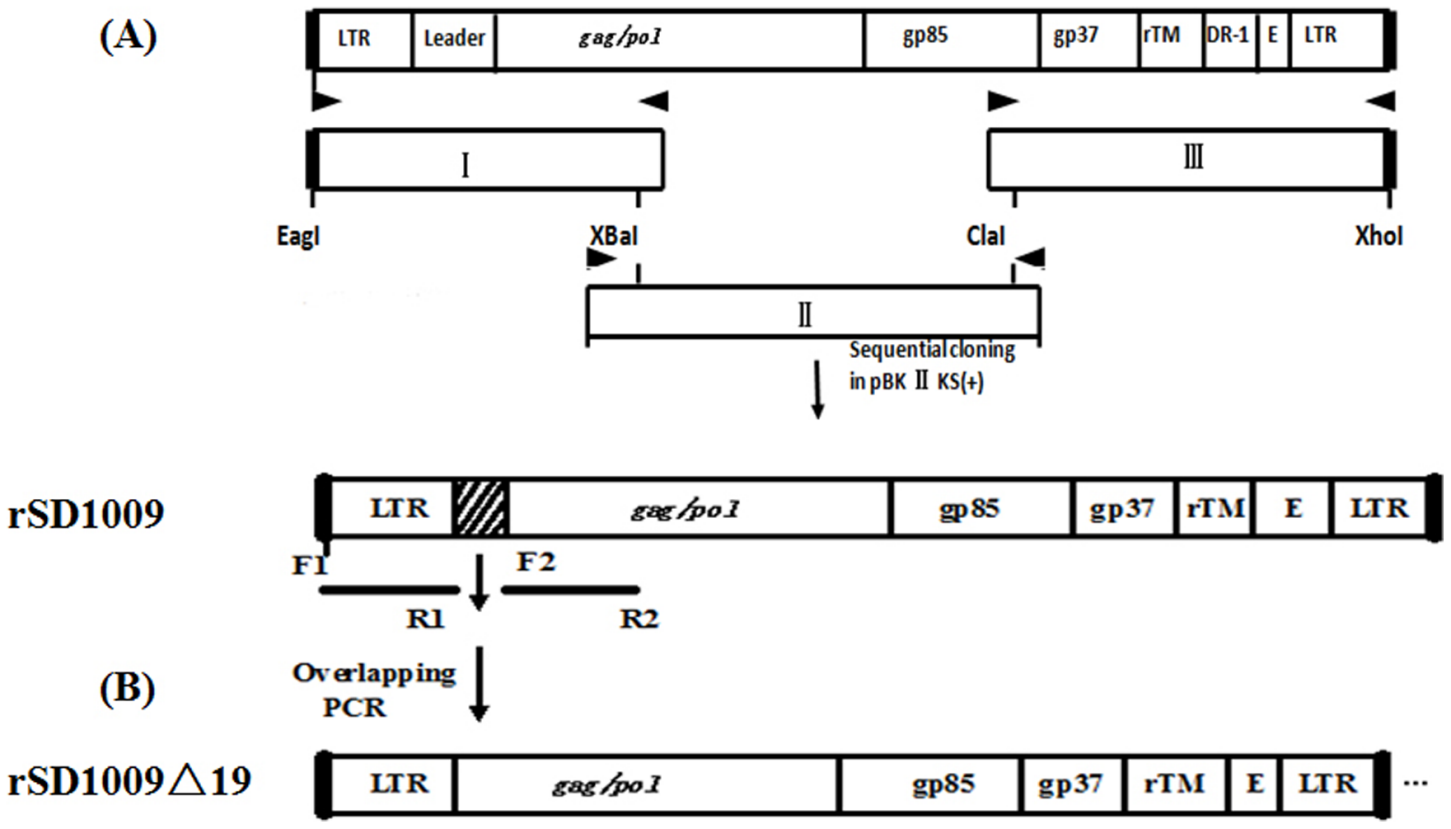
Schematic diagrams showing the construction of the rSD1009 and rSD1009△19 proviral DNA molecules. The structure of the SD1009 proviral genome (including the LTR, leader, *gag*-*pol*, *env* [gp85 and gp37], rTM, DR-1, and E element) is shown at the top. (A) The full-length rSD1009 proviral genome was assembled into the pBlueScript II KS (+) plasmid from subgenomic DNA fragments (I–III) that were generated by high-fidelity PCR (not to scale) to construct the rSD1009 proviral DNA molecules. The black arrows indicate the PCR primers. (B) The 19-nt deletion in the leader sequence was constructed as the rSD1009△19 proviral DNA.

5′-CCCTCGAGTGAAGCCATCCGCTTCATGCAGGTGCTCGTAGTTGTCAGG-3′ (downstream, position 7802–7841). The amplicons were gel purified and subcloned into pMD-18T vectors (TaKaRa Biotechnology Co. Ltd., Dalian, China) and ligated in order into pBlueScript II KS(+) plasmids (TaKaRa Biotechnology Co. Ltd.) via restriction enzyme digestion. The recombinant plasmid was named pBlu-SD1009. To delete the 19-nt insertion in the leader sequence, fragment I was replaced with a fusion product of two PCR fragments containing overlapping sequences spanning the deleted region. The primer sets were F1, 5′-CGAGCAGCCATCGATTTCTTACTC-3′ and R1, 5′-CTGGTGCGGACCACTCAGT-3′ and F2, 5′-ACTGAGTGGTCCGCACCAGGCGTGATTCTGGTCGCCCT-3′ and R2, 5′-CCCTCGAGTGAAGCCATCCGCTTCATGCAGGTGCTCGTAGTTGTCAGG-3′. Finally, the new fragments I, II, and III were ligated into the pBlueScript II KS(+) plasmid, in that order, to obtain the pBlu-SD1009△19 recombinant plasmid. The above construction for the two full-length cDNA clones is shown in Fig. 1.

### Viral rescue and identification

Highly purified pBlu-SD1009 and pBlu-SD1009△19 DNA was obtained using Qiagen Plasmid Midi Kits (Qiagen, Hilden, Germany) according to the manufacturer’s instructions. The purified plasmid DNA (4 µg) from pBlu-SD1009 and pBlu-SD1009△19 was introduced into DF-1 cells using Lipofectamine 2000 (Invitrogen, Carlsbad, CA, USA). The culture supernatant containing the viral stocks was harvested 48 h later and then blind-passaged into secondary DF-1 cells. The rescued viruses were named rSD1009 and rSD1009△19. To determine the specificity of the rescued viruses, the secondary DF-1 cell supernatants were analyzed using an enzyme-linked immunosorbent assay (ELISA; IDEXX, Inc., Westbrook, MA, USA) with the ALV group-specific antigen p27. An indirect immunofluorescence assay (IFA) was performed with virally infected DF-1 cells. Briefly, DF-1 cells were grown to 60% confluence in a tissue-culture plate and then infected with supernatants from the passage of rSD1009 and rSD1009△19. After incubation for 48 h at 37°C, the cells were washed once with phosphate-buffered saline/Tween 20 (PBST) and then fixed with ethanol for 20 min at room temperature. The fixed cells were incubated with monoclonal antibodies specific for ALV-J gp85 [14] in a humidified chamber for 60 min at 37°C and reacted with fluorescein-labeled goat anti-mouse immunoglobulin G (IgG) antibody (Sigma-Aldrich, St. Louis, MO, USA) in a humidified chamber for 60 min at 37°C. Next, the cells were washed with PBST and then examined by fluorescence microscopy. Non-infected DF-1 cells were used as a negative control and identification by electron microscopy was performed as described previously [15]. The HPRS-103 prototype virus was a generous gift from Prof. Venugopal Nair (Avian Viral Diseases Programme, BBSRC Institute for Animal Health, Compton, UK).

### Determination of the ALV-J titer

rSD1009, rSD1009△19 and HPRS-103 was inoculated into DF-1 cell culture bottles, freeze-thawed three times and then centrifuged at 7000×*g* and 4°C for 5 min. The supernatant was collected and 10-fold serially diluted to 10^−8^ and then inoculated into 96-well plates along with DF-1 cells. Eight parallel well were created in each gradient. After incubation for 5 days, the cells were washed once with PBST and then fixed with ethanol for 20 min at room temperature. The fixed cells were incubated with monoclonal antibodies specific for ALV-J gp85 in a humidified chamber for 60 min at 37°C and reacted with fluorescein-labeled goat anti-mouse immunoglobulin G (IgG) antibody (Sigma-Aldrich, St. Louis, MO, USA) in a humidified chamber for 60 min at 37°C. Next, the cells were washed with PBST. The number of fluorescent cells per well were counted using a fluorescence microscope, and the TCID50 was calculated using the Reed -Muench method [16].

### Reverse transcriptase (RT) activity and replication kinetics of the rescued viruses

RT activity and the replication kinetics in the culture supernatants of rSD1009, rSD1009△19, and HPRS-103 were measured. Each 60-mm-diameter plate of DF-1 cells (approximately 10^6^ cells/plate) was infected with approximately 0.1 ml of 10^2.5^/ml 50% tissue culture infectious doses (TCID_50_) of rSD1009, rSD1009△19, and HPRS-103. Post-infection culture supernatants were collected daily for 6 days and RT activity was quantitated using a colorimetric reverse transcriptase assay (Roche Applied Science, Indianapolis, IN, USA). To determine the replication kinetics of rSD1009, rSD1009△19, and HPRS-103 in more detail, DF-1 cells in 60-mm plates were infected with approximately 0.1 ml per plate of 10^2.5^/ml TCID_50_ of rSD1009, rSD1009△19, and HPRS-103, respectively. Infected cell cultures were harvested at various time points and the titers of infectious progeny were determined as TCID_50_ per milliliter using the Reed–Muench method as described above. Results are presented as mean values and standard deviations from three independent experiments.

### Luciferase-reporter plasmid construction

The luciferase-expression vector pGL3-Promoter Vector (Promega, Madison, WI, USA) was used as the parent vector for the leader sequences of rSD1009, rSD1009△19, and HPRS-103 reporter-analysis experiments. The leader sequences of rSD1009 (279 nts), rSD1009△19 (259 nts), and HPRS-103 (259 nts) were amplified by PCR. To facilitate cloning, *Kpn*I and *Xho*I restriction sites were added to the 5′ and 3′ primers. The primers used to amplify all leader sequences were as follows: forward, 5′-CCGGTACCCGTTAGGGAATAGTGGTCGGC-3′ and reverse, 5′-CCCTCGAGGCTTGATCCACAGGGCGACCA-3′. The PCR products were ligated to the pGL3-Promoter vector directly through gel retrieval (BioFlux, Hangzhou, China) and the constructs were named rSD1009-leader, rSD1009△19-leader, and HPRS103-leader after confirmation by sequencing.

### Pathogenicity analyses of the rescued viruses

One-day-old chicks of specific-pathogen-free (SPF) layers were purchased from the Experimental Animal Center of the Harbin Veterinary Research Institute, CAAS, China, and housed in negative-pressure-filtered air isolators. The animal experiments were approved by the Animal Ethics Committee of the institute. The SPF layers were infected on the day of hatching by intra-abdominal inoculation with approximately 0.25 ml of 10^2.5^/ml TCID_50_ of rSD1009 and rSD1009△19 viral stock per chicken. The titer used to inoculate the chickens in this study was based on a previous report that showed that similar titers could induce tumors [12]. Control chickens were injected with 0.25 ml of uninfected tissue culture fluid. Serum samples, collected from post-hatching birds 7 weeks post-infection, were analyzed using a microneutralization test as described previously [9]. The chickens were observed for 238 days and examined postmortem for any gross or microscopic tumors.

### Statistical analysis

The statistical significance was determined by Student’s t test using GraphPad Prism (version 5.0) software (GraphPad Inc., La Jolla, CA, USA).

## Results

### Viral isolation and identification

The ALV-J isolate SD1009, which was isolated from a layer chicken in the Shandong province in China [13], was used as the initial viral strain to infect DF-1 cells. Using primers H5 and H7, PCR amplification of DNA extracted from infected DF-1 cells produced an ALV-J-specific 545-bp fragment; however, the same DNA samples failed to produce specific fragments using primers specific to other viruses (data not shown). Further evidence of ALV-J in the samples was demonstrated by the positive results in the IFA using infected DF-1 cells with ALV-J-specific monoclonal antibodies (data not shown). These data indicated that ALV-J SD1009 was the pathogen associated with tumor growth in infected chickens.

### The 19-nt insertion in the leader sequence of ALV-J was isolated in China

**Figure 2:**
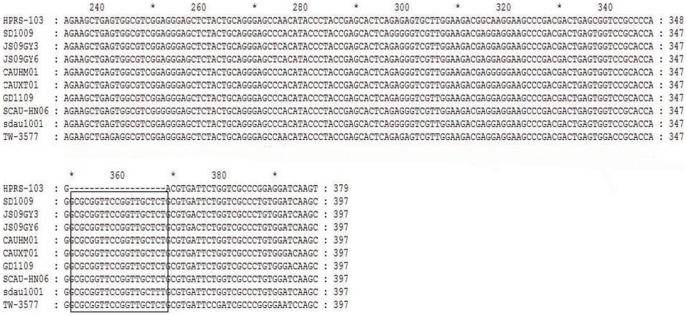
Sequence comparisons of the reference strains with the 19-nt insertion in the leader sequence. The 19-nt insertion in the leader sequence is enclosed in a black box.

The sequence of SD1009 was analyzed, which identified a unique 19-nt insertion in the leader sequence that was absent in the prototype ALV-J strain HPRS-103. To determine whether the 19-nt insertion in the ALV-J leader sequence was a common and new molecular characteristic of the ALV-J genome, the sequences of other ALV-J isolates were collected and analyzed. As shown in Fig. 2, a comparison of the leader sequences revealed the emergence of a unique, naturally occurring 19-nt insertion in the ALV-J genome from China (Fig. 2). A 19-nt insertion (5′-GCGCGGTTCCGGTTGCTCT-3′), located between nt 573 and 574 according to the HPRS-103 sequence, was present in the leader sequence. The leader sequence from 10 ALV-J isolates (including one classical ALV-J strain and nine Chinese isolates) were analyzed (Table 1).

**Table 1:** ALV-J strains used in sequence analysis**.**

Isolate	Origin	Yr	Accession no.	Host^a^
HPRS-103	UK	1988	Z46390	M
SD1009	China	2010	KF768000	CL
JS09GY3	Jiangsu, China	2009	GU982308	CL
JS09GY6	Jiangsu, China	2009	GU982310	CL
CAUHM01	China	2009	JF932000	C
CAUXT01	China	2009	JF932003	C
GD1109	China	2012	JX254901	C
SCAU-HN06	China	2007	HQ900844	CL
sdau1001	China	2010	JN389517	CL
TW-3577	Taiwan	2013	HM582657	C

a The host of the ALV-J isolates. CL, commercial laying chicken; M, meat-type chicken; C, chicken.

### Two viable viruses were rescued

**Figure 3:**
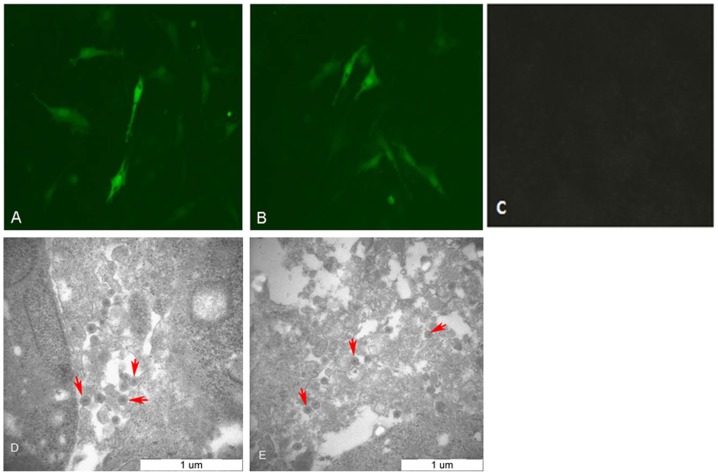
The identification of the rescued viruses by IFA and electron microscopy. (A) The DF-1 cells infected with rSD1009 using IFA mediated by monoclonal antibodies specific for ALV-J gp85 produced ALV-J-specific green fluorescence. (B) The DF-1 cells infected with rSD1009△19 using IFA mediated by monoclonal antibodies specific for ALV-J produced ALV-J-specific green fluorescence. (C) Uninoculated DF-1 cells served as a negative control. (D) Electron microscopy of the ultrathin sections of rSD1009-infected DF-1 cells revealed the virion. (E) Electron microscopy of ultrathin sections of rSD1009△19-infected DF-1 cells revealed the virion. The red arrows point to virion. Scale bar  =  1 µm.

Two viruses, rSD1009 (with the 19-nt insertion in the leader sequence) and rSD1009△19 (without the 19-nt insertion), were rescued using reverse genetics. An IFA was performed to identify the rescued viruses. The DF-1 cells infected with the rescued viruses were stained by monoclonal antibodies specific for ALV-J gp85. Primary antibody binding was detected using fluorescein isothiocyanate-labeled anti-mouse IgG, which exhibited green fluorescence (Fig. 3A and B). Electron microscopy revealed two viruses with diameters of approximately 90 nm in the ultrathin sections of rSD1009- and rSD1009△19-infected DF-1 cells (Fig. 3D and E). These data demonstrated that the rSD1009 and rSD1009△19 viruses were rescued.

### The 19-nt insertion in the leader sequence enhanced leader enhancer activity and viral replication in DF-1 cells

**Figure 4:**
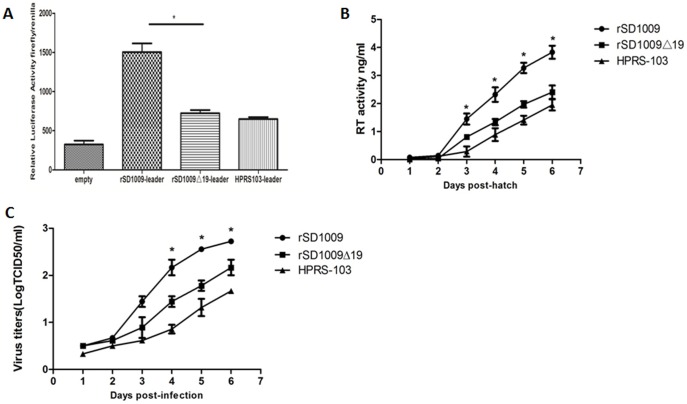
The 19-nt insertion in the leader sequence enhanced activity of the leader sequence and viral replication in DF-1 cells. (A) Comparison of enhancer activity of the leader sequence of rSD1009, rSD1009△19, and HPRS-103 in constructs expressing a firefly reporter gene. The Renilla luciferase gene was used as a reference gene for normalization of gene expression in transiently transfected cells. (B) The results of the RT assays performed on the virus particles isolated from the culture supernatants of DF-1 cells infected with each virus are shown. The growth curves were drawn by RT assays. (C) One-step growth curves for rSD1009, rSD1009△19, and HPRS-103 in DF-1 cells. The growth curves were drawn by assaying the viral titers. The viral titers harvested at different intervals were calculated and expressed as TCID_50_ per milliliter. The standard deviations (error bars) from three independent experiments are shown. Significant differences between the rSD1009 and rSD1009△19 groups are indicated by asterisks (*, *p* < 0.05). Statistical analysis was performed by Student’s t test.

To explore whether the 19-nt insertion in the leader sequence influenced the enhancer activity of the leader sequence, we constructed a luciferase vector with a leader sequence with and without the 19-nt insertion. We also constructed a luciferase vector with the same leader sequence as HPRS-103. As shown in Fig. 4A, the results indicated a significant increase in the leader sequence constructs with the 19-nt insertion, which suggested that the 19-nt insertion in the leader region enhanced the leader enhancer activity.

To further explore whether the 19-nt insertion in the leader sequence conferred a growth advantage to the virus, DF-1 cells, which are commonly used for ALV-J proliferation [17], were used to evaluate *in vitro* replication of the rescued virus. DF-1 cell cultures were infected with equivalent amounts of rSD1009, rSD1009Δ19, and HPRS-103. The culture supernatants were harvested periodically thereafter for reverse transcriptase (RT) activity assays. For the first 2 days, rSD1009 displayed growth curves similar to rSD1009Δ19 before the RT activity could be detected. Subsequently, rSD1009-infected cells produced more viral particles than those infected with rSD1009△19. By day 6, the number of viral particles of both strains peaked. The results revealed that rSD1009 had a moderate growth advantage over rSD1009Δ19 (Fig. 4B), suggesting that the 19-nt insertion enhanced ALV-J replication in DF-1 cells. Although RT activity can be used to determine retroviral replication [18], direct titration was performed to confirm replication of rSD1009, rSD1009Δ19 and HPRS-103. As shown in Fig. 4C, the direct titration of rSD1009 and rSD1009Δ19 was similar to the RT activity results and the 19-nt insertion-enhanced viral replication in DF-1 cells.

### rSD1009 and rSD1009Δ19 have similar replication and pathogenicity *in vivo*

To evaluate *in vivo* replication and pathogenicity of the rescued viruses, 1-day-old SPF layer chicks were intra-abdominally inoculated with rSD1009 and rSD1009. Compared to controls, most of the infected chickens gradually became emaciated as they grew and some became paralyzed by 3 months post-infection, which is a classical phenomenon in ALV-J-infected chickens [19].

To compare the pathogenic effects of the rescued viruses, infected chickens were observed for 238 days. Evidence of viral replication was apparent in these chickens via examination of serum samples 7 weeks post-infection [9], which confirmed neutralizing antibodies in 4/12 (33.33%) of the rSD1009-infected and 4/13 (30.76%) of the rSD1009△19-infected chickens. No significant differences in viral replication were observed between the rSD1009 and rSD1009△19 infection groups. These data demonstrated that the 19-nt insertion did not influence ALV-J replication *in vivo*.

**Figure 5:**
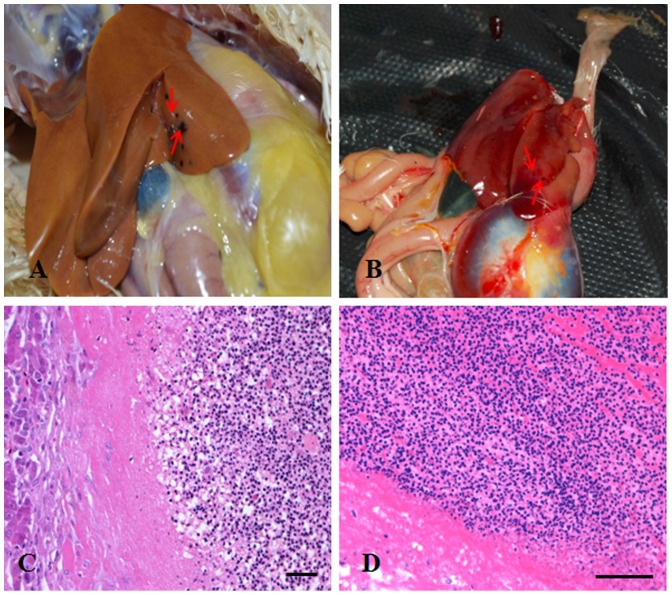
Clinical manifestations and histological examinations of tumors from chickens infected with rSD1009 and rSD1009△19. (A) The liver of an SPF layer infected with rSD1009. (B) The liver of an SPF layer infected with rSD1009△19. The red arrows point to clinical lesions. (C) Histological examination of (A) liver; a large, well-demarcated, non-encapsulated nodule consisting of numerous erythroblasts (as shown on the right side). (D) Histological examination of (B) liver; a large, well-demarcated, non-encapsulated nodule consisting of numerous erythroblasts. All histological examinations used hematoxylin and eosin stain. Scale bar  =  50 µm.

Levels of oncogenicity were determined from the incidence of histological tumors. In chickens infected with either of the viruses, there was only one chicken with an induced tumor. There was no difference in the incidence of tumors between chickens infected with rSD1009 (8.3%) and rSD1009△19 (7.6%). Details regarding the types of identified tumors are shown in Table 2. The clinical manifestations of the rSD1009- and rSD1009△19-induced tumors and the histological findings of those tumors (diagnosed as erythroblastosis) are shown in Fig. 5. PCR detection and sequencing also confirmed that all chickens with tumors had infections with the inoculated viruses (data not shown). Uninfected control chickens displayed no tumors.

**Table 2:** In vivo infection and tumorigenesis in rSD1009- and rSD1009△19-infected groups

Virus	Age at infection	Infection status*(%)	Details of tumors	
SPF layer chicken			SPF layer chicken	
			Tumor incidence(%)	Tumor of type(n)
rSD1009	1-day-old chick	4/12(33.33)	1/12(8.33)	EB(1)
rSD1009△19	1-day-old chick	4/13(30.76)	1/13(7.69)	EB(1)
Uninfected control	1-day-old chick	0/13(0)	0/13(0)	NA

* Infection status is based on the results of neutralizing antibodies of serum samples 7 weeks after infection. NA, not applicable; erythroblastosis (EB).

## Discussion

Since 2008, ALV-J infections in chickens have become widespread in China [6], [20]. The genomic sequences of ALV-J epidemic isolates compared with HPRS-103, the ALV-J prototype virus, exhibits several distinct features. In this study, a unique 19-nt insertion in the leader sequence was identified. Thus, we investigated the function of the 19-nt sequence both *in vitro* and *in vivo* by construction and rescue of a pair of viruses (rSD1009 and rSD1009△19). The results indicated that the 19-nt insertion in the leader sequence of ALV subgroup J contributed to its replication *in vitro*, but was not related to its pathogenicity *in vivo*.

It is important to monitor sequence changes in the leader sequence of ALV-J because the 5' leader sequences of avian retroviruses contain functional elements involved in key steps of the retroviral life cycle [21]. A previous report indicated that ALV-J was prone to mutations that facilitate the relatively rapid evolution of the virus [12]. A 19-nt insertion, observed in the leader sequence of ALV-J in this study, was naturally occurring, suggesting that it was the result of the natural evolution of the ALV-J genome.

We employed DF-1 cells, which are commonly used for propagating ALV-J [22], to evaluate *in vitro* replication of the rescued viruses. Our results indicated that rSD1009 replicated more rapidly than rSD1009△19 in DF-1 cells (Fig. 4B and C). In addition, we also confirmed that the leader sequence with the 19-nt insertion enhanced protein expression using a luciferase assay (Fig. 4A). The ALV-J leader sequence has three unique open reading frames, which may be positioned to influence viral activities, such as expression of viral proteins [23]. The 19-nt insertion was found to influence viral replication *in vitro*, which may be the reason that the insertion changed the length of the leader sequence, thereby re-adjusting its structure and ultimately influencing the cis-acting characteristics of the leader sequence in the ALV-J strain.

Although the 19-nt insertion enhanced *in vitro* replication, it did not appear to affect viral replication *in vivo*, as demonstrated by the neutralizing-antibody response of infected chickens at 7 weeks of age (Table 2). This discrepancy between viral replication *in vitro* and *in vivo* has also been observed in other viruses, such as a single amino acid mutation in very virulent infectious bursal disease virus that could attenuate replication *in vivo* but increased replication *in vitro* [24]. The mechanism explaining why the 19-nt insertion had different effects on ALV-J replication *in vitro* and *in vivo* requires further investigation.

There was no significant difference in the prevalence of tumors in chickens following infection with either rSD1009Δ19 or rSD1009. In addition, the oncogenic spectrum of the two viruses was very similar (Table 2). The oncogenicity of ALV-J is an important index of its pathogenicity [9], [12]. These data strongly suggest that the 19-nt insertion in the leader sequence was not related to the pathogenicity of ALV-J in SPF layers, which is in agreement with the similar replication levels of rSD1009 and rSD1009△19 *in vivo*. A previous hypothesis suggested a link between *in vivo* replication and pathogenicity and proposed that viral replication and pathogenicity were positively correlated [25]. Here, the similar *in vivo* replication of rSD1009 and rSD1009△19 led to similar pathogenicity to the SPF layers, supporting the proposed hypothesis. Although a correlation between pathogenicity and effective viral replication has also been observed in other reports, including a study that linked the replication of Newcastle disease virus with its pathogenicity [26] and a second that revealed increased pathogenicity of avian influenza viruses with efficient viral replication [27], the molecular mechanisms mediating the relationship between the viral replication and pathogenicity remain unclear.

ALV-J-induced disease among chicken flocks in China has been surprising and generated a large amount of attention worldwide [5], [6], [28], [29], [30]. In the present study, molecular epidemiological research revealed that the 19-nt insertion, naturally occurring in ALV-J isolates from China, was a newly added molecular characteristic to the ALV-J genome. Using reverse genetics and animal experiments, here, we report the first evidence that the 19-nt insertion in the leader sequence of ALV-J contributed to the increased replication of ALV-J *in vitro*, but was not related to its pathogenicity *in vivo*. This study not only demonstrated a unique molecular characteristic of ALV-J, but also generated data that may benefit the understanding of the relationships between variations in genomic sequences and the oncogenicity of ALV-J.
